# A psychological reaction of higher education students to the new national exit exam in the case of Dilla University, 2023

**DOI:** 10.3389/fpsyg.2024.1431707

**Published:** 2024-11-15

**Authors:** Chalachew Kassaw, Valeriia Demareva, Samrawit Getu, Misrak Negash, Selamawit Alemayehu

**Affiliations:** ^1^Department of Psychiatry, Dilla University, Dilla, Ethiopia; ^2^Department Cyber Psychology, Lobachevsky University, Nizhniy Novgorod, Russia; ^3^Department of Psychiatry, St. Paul Hospital Millennium Medical College, Addis Ababa, Ethiopia

**Keywords:** test anxiety, national exit exam, higher education students, prospective graduates, Dilla University, Ethiopia

## Abstract

**Introduction:**

Test anxiety is an emotional state characterized by physiological and behavioral responses linked to the fear of poor exam results. It can result in a significant impact in the overall academic achievement of students. Addressing the contributing factors of the problem is better to intervene in the academic challenges of students and create a conducive learning environment. Thus, this study investigated the association between test anxiety and the newly implemented national exit exam program among prospective graduate students at Dilla University in 2023.

**Methods:**

A community-based cross-sectional design was employed, involving 357 participants recruited from August 15 to September 14, 2023. Data collection utilized structured questionnaires combined with interviews. The Westside Test Anxiety Inventory (WTAI) assessed test anxiety levels. Epi Info version 7 facilitated data entry, with subsequent analysis conducted using SPSS version 25. A *p* < 0.05 was used to determine statistical significance in the multivariate logistic regression model.

**Results:**

The prevalence of test anxiety associated with the national exit exam among prospective graduates at Dilla University was 68.9% (95% CI: 63.9–73.7%). The analysis identified significant associations between test anxiety and several factors: non-formal educational background of parents (AOR = 2.84, 95% CI: 2.12–4.65), moderate social support (AOR = 0.24, 95% CI: 0.12–0.46), and poor coping mechanisms (AOR = 3.67, 95% CI: 2.45–5.67).

**Conclusion:**

This study revealed a substantial prevalence of test anxiety among graduating students about the national exit exam. The findings suggest that factors such as limited social support and inadequate coping mechanisms contribute to test anxiety. Targeted interventions, including social support programs, counseling services, coping mechanism training, parental education, and support for students with lower academic performance, may be beneficial in mitigating test anxiety.

## Introduction

Test anxiety is characterized as an unpleasant emotional state with physiological and behavioral implications linked with fear of poor exam performance. It is a prevalent condition across students from numerous fields (Quinn and Peters, 2017; Soares and Woods, 2020). It develops as a mix of mental, bodily, and behavioral reactions induced by the dread of underperforming on tests (Hamzah et al., 2018). The course of the condition can manifest suddenly or gradually and varies in duration. The exhibited symptoms include loss of appetite, the difficulty of sleep, sweaty palms, and difficulty in concentration (Balasuburamaniam, 2019).

Test anxiety can both motivate and hinder exam performance, as it enhances focus and alertness, promoting thorough exam preparation (Spielberger, 2021). Excessive test anxiety can lead to physiological symptoms like difficulty concentrating and impaired cognitive function, significantly hindering exam performance (Pate et al., 2021), This can lead to students forgetting previously learned information or struggling to apply their knowledge effectively during exams (Tsegay et al., 2019). Test anxiety can be both beneficial and detrimental to exam preparation and performance, depending on its severity (Hill and Wigfield, 1984). Test anxiety prevalence in universities worldwide is unclear, possibly due to departmental factors like coursework intensity, with high-stakes fields like medicine and engineering experiencing higher prevalence (Macaro and Han, 2020). Departmental assessment styles, student demographics, and learning styles may influence test anxiety rates, with standardized exams potentially fostering more anxiety than diverse projects or presentations (Oladipo and Ogungbamila, 2013a).

Previous studies on test anxiety prevalence across different countries reveal significant geographical variations. Studies report a range of 25–40% in the USA (Hanfesa et al., 2020) to a staggering 89.4% in the United Arab Emirates (Tassadaq et al., 2016). Similarly, estimates in China vary between 27 and 35.7% (Liu et al., 2021), while India displays a considerably higher rate of 83% (Manisha et al., 2019). Other studies report prevalence at 38.5% in Canada (Gerwing et al., 2015), 32.6% in Malaysia (Rajiah et al., 2014), 48% in Turkey (Kavakci et al., 2014), 65% in Saudi Arabia (Khoshhal et al., 2017), 43.4% in Iran (Asayesh et al., 2016), 35% in Sudan (Bashir et al., 2019) and 68.1% in Kenya (Ndirangu et al., 2009). Additionally, within Ethiopia, studies at Gondar and Addis Ababa Universities documented rates of 54.7% (Hanfesa et al., 2020) and 52.3% (Tsegay et al., 2019). Previous studies have identified numerous factors associated with test anxiety. These include demographic characteristics such as female gender and lower age, academic factors like low grade point average and first-year student status, and psychological factors such as low self-esteem and ineffective time management (Alamri and Nazir, 2022; Markman et al., 2010; Oladipo and Ogungbamila, 2013b). Additionally, heavy course loads, lack of social support, sleep disturbances, and social media usage have been linked to heightened levels of test anxiety (Chukwuorji and Nwonyi, 2015). Recommended solutions to address test anxiety include effective coping strategies such as problem-focused coping, emotion-focused coping, prayer, and seeking social support.

In Ethiopia, there have been expansions of public and private higher education institutions from time to time. However, the quality of education is still a problem, and there is a need for intervention at various levels of the education system. The Ethiopian Ministry of Education introduced a nationwide exit examination in 2022/2023 for graduate students, aiming to set minimal knowledge and skill benchmarks across all Ethiopian universities. The national exit exam aims to address concerns about undergraduate education quality and graduates’ preparedness for postgraduate studies by assessing student learning outcomes, identifying shortcomings, and providing valuable data for improvement. System-wide assessment is crucial for higher education institutions to meet pressure and fulfill missions. It is also useful to change student assessment practices. Strategic assessment can motivate students, inform staff, and provide performance indicators. One way to achieve this is by introducing high-stakes, standardized exit exams. These exams encourage students to achieve their best and provide teachers with high-level instruction. Exit examinations serve various purposes, including raising awareness about public accountability, providing feedback, and demonstrating students’ skill acquisition (Abie et al., 2023; Asemu et al., 2023b). Despite the importance of this national competency exit exam on the quality of education, there might be undesirable emotional and psychological reactions from a student’s perspective. This study investigates factors contributing to test anxiety among Dilla University graduates, aiming to provide insights into the anxiety associated with the first national exit exam in 2023. The findings can inform targeted interventions and support systems to optimize student wellbeing and performance.

## Materials and methods

### Study area and period

The study took place at Dilla University, situated in the Gedeo zone within the Dilla city administration of the Southern Nations and Nationalities Region (SNNR), Ethiopia. It is located at a distance of 365 km from the capital city, Addis Ababa. Data collection occurred within Dilla University, which comprises three campuses: the main campus, Odaya campus, and the health campus. The university consists of four colleges: Natural Science and Computational Science, Engineering and Computer Science, Medicine and Other Health Sciences, and Social and Humanities Science College. The study was conducted from August 15 to September 14, 2023 encompassing approximately 2000 graduating students at the university. The Ethiopian Ministry of Education’s implementation of an exit exam for all undergraduate students signifies a significant stride toward enhancing the quality of higher education. This initiative aims to prepare graduates for success in the job market and to contribute effectively to the nation’s development (Asemu et al., 2023a).

### Study design

This study used an institutional-based cross-sectional design.

### Population

*Source of population:* All prospective graduate students at Dilla University.

*Study population*: All Dilla University prospective graduate students who were contacted during the data collection period.

*Study unit:* Students who were interviewed during data collection.

### Eligibility criteria

*Inclusion criteria:* All prospective graduate students who were eligible to be interviewed were included in the study.

*Exclusion criteria:* Prospective graduate students who had acute physical or mental conditions and were unable to give information during the data collection period were excluded from the study.

### Sample size determination

The sample size was determined using a single population formula, by considering the magnitude of test anxiety related to the exit exam considering the following assumption: The previous study conducted at Addis Ababa University was selected to calculate the current sample size. This decision was made because the previous study aligns with the current study’s research questions and hypothesis, shares a similar outcome variable, and employed reliable and validated assessment tools.


$$n=\frac{\left(Z\alpha /2\right)2\;p\;\left(1-p\right)}{d^{2}}$$

Where *n* = minimum sample size required for the study.

Z = standard normal distribution (Z = 1.96) with confidence interval of 95% and *⍺* = 0.05.

P = the prevalence of test anxiety in Addis Ababa University Ethiopia is 52% (Tsegay et al., 2019). Hence, *p* = 0.52 was used.

d = Absolute precision or tolerable margin of error (d) = 5% = 0.05.

Therefore, the sample size calculated was 383 with a non-response rate of 10% (Saravanan et al., 2014), the final sample size was 421.

### Sampling technique

**Figure 1:**
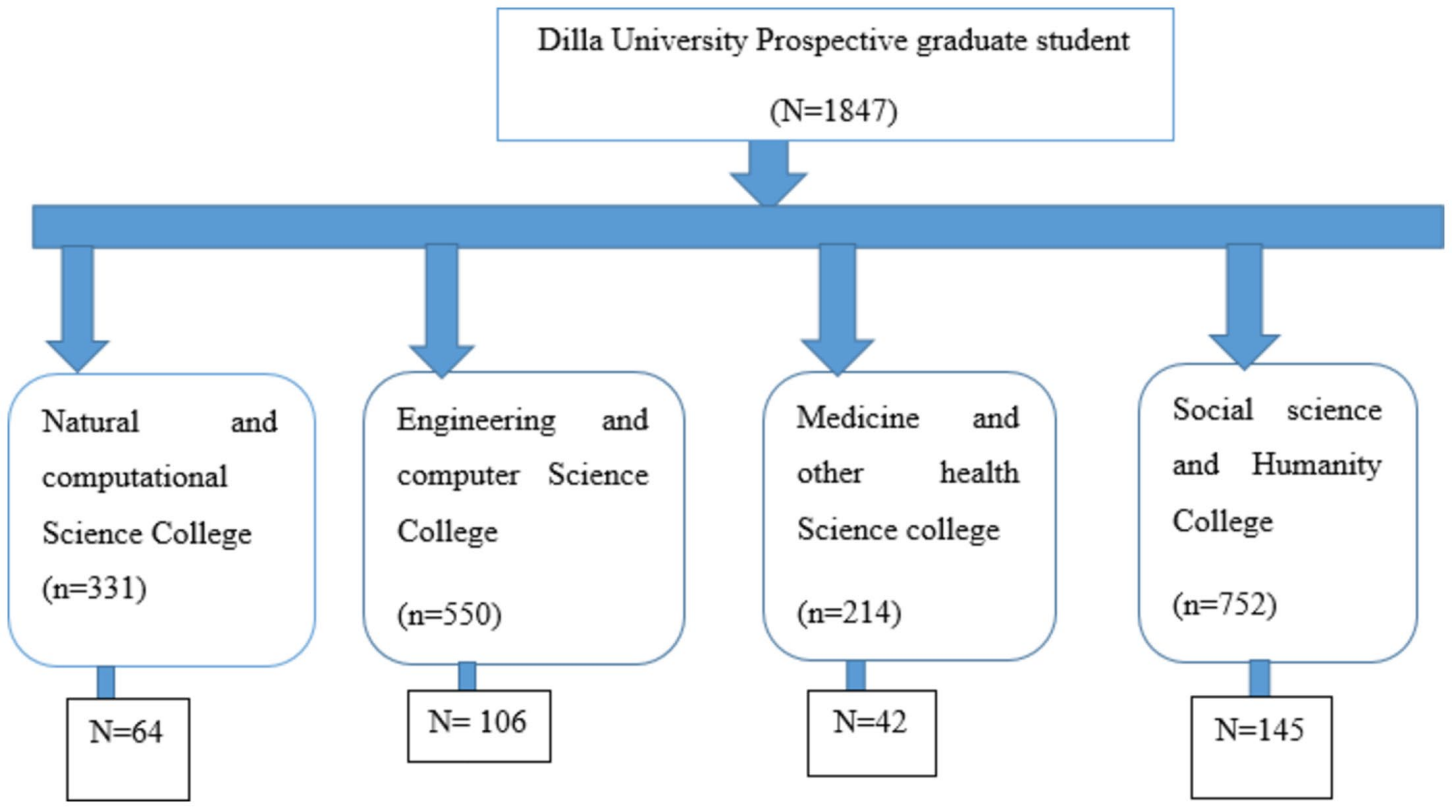
Schematic presentation of sampling technique (*n* = 357).

The study utilized a systematic random sampling technique to select participants. Dilla University’s annual reports indicate approximately 1847 prospective graduates across its colleges: Natural and Computational Science (*n* = 331), Engineering and Computer Science (*n* = 550), Medicine and Other Health Science (*n* = 214), and Social and Humanities College (*n* = 752). Systematic random sampling was employed to select individual samples. The kth interval was determined by dividing the total student size in each college by the total sample size, where N represents the total number of students in each college, and n denotes the total sample size. Participants were selected using the systematic random sampling technique at regular kth intervals during the data collection period. The study involved a proportional allocation of study participants across the four colleges. To calculate the Kth Interval, we used N/*n* = 1847/421 = 4th (Figure 1).

### Study variable

#### Dependent variable

Test anxiety.

#### Independent variable

*Sociodemographic characteristics:* age, sex, education level, occupation, monthly income, marital status.

*Academic factors*: GPA, year of study, college of study, and department.

*Psychosocial factors:* social support, psychological distress, sleep quality, coping mechanism, and substance/internet use.

### Measures for the dependent variable (test anxiety)

*Westside Test Anxiety Inventory (WTAI):* Data for the dependent variable (test anxiety) were collected using a self-administered questionnaire. The questionnaire used was the Westside Test Anxiety Inventory (WTAI), developed by Driscoll (2007). It consisted of 10 statements, and respondents were asked to indicate how often they experienced anxiety symptoms before, during, and after taking tests. Each statement was scored using a 5-point Likert scale, resulting in a total test anxiety score ranging from 10 to 50 points. Respondents were categorized into six levels of test anxiety: comfortably low-test anxiety (score of 1.0–1.9), normal or average test anxiety (score between 2.0 and 2.5), high normal test anxiety (score between 2.5 and 2.9), moderately high-test anxiety (score between 3.0 and 3.4), high-test anxiety (score between 3.5 and 3.9), and extremely high anxiety (score between 4.0 and 5.0). The cut-off point for problematic test anxiety was set at ≥30. Therefore, as per this study, respondents who scored below 30 were classified as having no test anxiety, while those who scored 30 and above were considered to have test anxiety. The Westside Test Anxiety Scale is an extremely brief screening instrument meant to identify students with anxiety impairments. The scale is comprised of 10 items, and takes about 5–8 min to administer. The Test Anxiety Inventory was designed to measure anxiety progress in tests examinations and to measure test anxiety level of an individual in evaluative situations. The 10-item inventory is designed to assess two components of test anxiety: Worry and, Emotionality. The reliability of the WTAI questionnaire was assessed using Cronbach’s alpha, which yielded a score of 0.87. Additionally, a split-half reliability of 0.82 was found in a Nigerian sample (Tsegay et al., 2019; Driscoll, 2007; Talwar et al., 2019).

### Measures for the predictor variables

#### The sociodemographic and other variables

Self-reported data on sociodemographic and other characteristics of the respondents were gathered using structured questioners.

#### Oslo social support

It is used to assess the level of social support among the respondents. The scores ranged from 3 to 8 indicating poor social support, 9–11 indicating moderate social support, and 12–14 indicating strong social support. The OSSS-3 is a concise and cost-effective scale with satisfactory internal consistency, as indicated by a reliability coefficient (*α*) of 0.64. Those who are found to score on Oslo −3 scale 3–8 was considered as having Poor support, 9–11 as having moderate support, and a score of 12–14 as having strong support (Kocalevent et al., 2018). In this study, the Assessment tool showed good consistency and Cronbach alpha 0.81.

#### The Pittsburgh sleep quality index (PSQI)

A self-report measure comprising 19 items, was utilized to assess maternal sleep quality over the past month. This tool was employed with a cutoff value set at >5. The diagnostic sensitivity and specificity of the outcome variable measurement were found to be 82 and 56.2%, respectively. The PSQI encompasses seven component scores, measuring various aspects such as subjective sleep quality, sleep latency, sleep duration, sleep efficiency, sleep disturbances, use of sleeping medication, and daytime dysfunction. These component scores range from 0 to 3 and are combined to generate a global PSQI score, ranging from 0 to 21. Higher scores indicate a higher level of overall sleep disturbances. A global PSQI score equal to or exceeding 5 is indicative of poor sleep quality (Nelson et al., 2022).

#### Grade point average (GPA)

It is a summary statistic that represents a student’s average performance in their studies over a stated period of time such as one semester (Sadler, 2023).

#### Kessler psychological distress scale (K-10)

It was assessed for measuring psychological distress. The K-10 scale comprises 10 questions regarding emotional wellbeing, each with a response scale consisting of five levels. The scale categorizes individuals based on scores: 10–19 suggests a state of wellness, 20–24 implies a mild disorder, 25–29 indicates a moderate disorder, and 30–50 suggests severe distress. This scale demonstrates a sensitivity rate of 70% and a specificity rate of 67% (Andersen et al., 2011).

#### Coping mechanism

The assessment of coping techniques employed by participants utilized the Academic Stress Coping Style Inventory. This inventory demonstrated a Cronbach’s alpha of 0.83, indicating strong internal consistency. The instrument was refined during the content validation phase, resulting in a 25-item Likert-scale questionnaire (1 = strongly disagree, 5 = strongly agree). Overall stress coping was categorized based on cumulative scores: 25–58 = poor coping, 59–92 = average coping, and 93–125 = good coping (Joseph et al., 2021).

### Data collection tool and procedures

To ensure the high quality of the data, the data collectors in this study underwent comprehensive training on data collection procedures and protocols before engaging in the actual data collection activities. All of the data collectors received the information from respondents together from each college. The data collection process involved the utilization of a self-administered questionnaire in English version. This questionnaire consisted of various components that assessed different aspects of the participants. The first part focused on gathering socio-demographic characteristics of the participants. The second part aimed to measure the level of test anxiety using the WTAI scale. The third part examined psychosocial factors (including psychological distress, coping mechanisms, and social support) that influence the level of test anxiety and were measured using the K-10 scale, Academic stress coping style inventory, and Oslo 3- Social Support Scale, respectively. The fourth part explored academic-related factors that influence test anxiety through the utilization of yes or no questions.

### Data quality control

To ensure data quality, the research team employed a rigorous data collection process. Following the development of an appropriate questionnaire, the instrument was further refined based on feedback. Training sessions equipped data collectors and supervisors with the necessary skills. A pre-test involving 18 students from Info Link Private University College was conducted 2 days before the main data collection to assess the questionnaire’s clarity and identify any logistical issues. Pre-test data was excluded from the final analysis. Based on the pre-test findings, the questionnaire underwent revisions, and an estimated timeframe for data collection was established. To ensure data quality, all of the data collectors went in each college to receive information from respondents. Daily supervision ensured smooth data collection, with supervisors addressing any problems encountered by data collectors through open discussions.

### Data processing and analysis

Following data cleaning to ensure completeness, statistical analyses were conducted using SPSS version 25. Associations between independent variables and test anxiety were explored using both unadjusted (crude) and adjusted odds ratios. For categorical independent variables, chi-square tests were employed to assess the assumptions of the chosen statistical models. Frequency tables, charts, and graphs were utilized to present the study’s findings. Before analysis, the normality of continuous data was evaluated. Descriptive statistics were reported accordingly, with means and standard deviations presented for normally distributed data, and medians with interquartile ranges reported for skewed data. Given the dichotomous nature of the outcome variable (Yes or No for test anxiety), a logistic regression analysis model was employed to examine the relationships between independent and dependent variables. Variables identified as statistically significant in bivariate analyses (*p* ≤ 0.025) were entered into the multivariable model for further evaluation. In the final multivariable model, a *p* < 0.05 was considered statistically significant for the association between a variable and test anxiety.

## Results

### Socio-demographic characteristics of respondents

The study had a high response rate of 85%, with a young population of 357, predominantly female (53.8%), and most religious affiliation toward Christianity. Marital status was skewed toward singles (90.8%), and most respondents lived in urban areas. The average distance from home was 472 km, and the monthly income averaged 1514 ETB. Over 70% had both parents alive, with half having formal education. Most parents were involved in private or merchant work, followed by government employment and farming (Table 1).

**Table 1:** Sociodemographic characteristics of respondents at Dilla University, 2023 (*n* = 357).

Variables	Category	Frequency	Percent
Sex	Male	165	46.2%
	Female	192	53.8%
Religion	Muslim	87	24.4%
	Orthodox	140	39.2%
	Protestant	130	36.4%
Marital status	Married	33	9.2%
	Single	324	90.8%
Resident	Rural	84	23.5%
	Urban	273	76.5%
Presence of father and mother	Both are alive	251	70.3%
	Either mother or father dead	106	29.7%
Educational status of parents	No	165	46.2%
	Yes	192	53.8%
Parents occupation	Farmer	50	14%
	Government employee	96	26.9%
	Private work or merchant	211	59.1%
Income	Low income (<1514)	247	69.2%
	High income (>1514)	110	30.8%
Sibling	>4	120	33.6%
	< 4	237	66.4%
Distance from home	<472 KM	217	60.8%
	>472 KM	140	39.2%

### Academic characteristics

The study involved 357 respondents from various colleges, with the majority in their fourth year. The largest group had a GPA between 2.5 and 3.0, followed by those with a GPA of 3.0–3.5 and those with a GPA between 3.5 and 4 (Table 2).

**Table 2:** Academic related factors of respondents at Dilla University, 2023 (*n* = 357).

Variable	Category	Frequency	Percentage
College attended	Computational and natural science	64	17.9%
	Engineering and computer science	108	30.3%
	Health science	42	11.6%
	Social science	143	40.1%
Grade point average (GPA)	<2.5	29	8.1%
	2.5–3	146	40.9%
	3–3.5	119	33.3%
	3.5–4	63	17.6%
Year of study	4	237	66.4%
	5	113	31.6%
	7	7	2.0%

### Psychosocial characteristics of participants

The study found a high prevalence of psychological distress and poor sleep quality among students, with an average of 5 h of sleep per night. Over half reported good coping mechanisms, while 43.7% lacked effective strategies. Social support was primarily moderate (75.6%), with some individuals experiencing lower levels (19.6%). Social media use was widespread (93.8%), averaging over 3 h daily. Substance use was present in 22.7% of participants, with alcohol (14.6%) and khat (7.0%) being the most commonly used substance (Table 3).

**Table 3:** Psychosocial factors of respondents at Dilla University, 2023 (*n* = 357).

Variable	Category	Frequency	Percentage
Psychological distress	Yes	351	99.3%
	No	6	1.7%
Sleep quality	Good	153	42.9%
	Poor	204	57.1%
Coping mechanism	Good	201	56.3%
	Poor	156	43.7%
Social support	Poor	70	19.6%
	Moderate	270	75.6%
	Strong	17	4.8%
Substance use	No	276	77.3%
	Yes	81	22.7%
Type of substance	Alcohol	52	14.6%
	Khat	25	7.0%
	Tobacco	4	0.9%
Presence of mental illness	No	324	90.8%
	Yes	33	9.2%
Social media use	No	22	6.2%
	Yes	335	93.8%
Presence of medical diagnosis	No	298	83.5%
	Yes	59	16.5%

### Prevalence of problematic test anxiety related to national exit exam among prospective graduate students

The current study identified a high prevalence of problematic test anxiety among participants, with 68.9% reporting symptoms within the concerning range. This finding is further substantiated by the 95% confidence interval, which extends from 63.9 to 73.7%, indicating a statistically significant level of problematic test anxiety within the study population. In our sample, the internal consistency of the WTAI was found to be very high, with a Cronbach’s alpha of 0.94.

### Binary logistic regression analysis result

#### Factors associated with test anxiety related to national exit exam among prospective graduate students

In this study, several factors emerged as significantly associated with test anxiety. Respondent’s parents with a non-formal educational background were 2.84 times (AOR = 2.84, 95% CI: 2.12–4.65), more likely to experience test anxiety as compared to their counterparts. Respondents with moderate social support had a 76% reduced odds of developing test anxiety (AOR = 0.24, 95% CI: 0.12–0.46). Academic performance also played a role, respondents with grade point average between 2 and 3 were 3.25 times more likely to experience test anxiety (AOR = 3.25, 95% CI: 2.45–6.78). Finally, Respondents who reported poor coping mechanisms exhibited 3.67 times more likely to develop test anxiety (AOR = 3.67, 95% CI: 2.45–5.67). The final model demonstrated a strong explanatory power (Cox and Snell R-square of 76%), and the null hypothesis of the Hosmer-Lemeshow test (*p* = 0.28) indicated good model fit (Table 4).

**Table 4:** Factors associated with test anxiety among respondents at Dilla University, 2023 (*n* = 357).

Variables	Category	Test anxiety		COR	Sig	AOR	Sig
		Yes	No				
Educational status of parents	Non-informal	122 (49.6%)	43 (38.7%)	5.15 (3.28–8.17)	0.02	2.84 (2.12–4.65)	**0.03**
	Formal	68 (61.3%)	124 (50.4%)	1		1	
Coping mechanism	Good	56 (35.6%)	145 (72.5%)	1		1	
	Poor	101 (64.3%)	55 (27.5%)	4.75 (3.03, 7.46)	0.01	3.67 (2.45–5.67)	**0.02**
Social support	Poor	41 (16.7%)	29 (26.1%)	3.39 (1.08–10.6)	0.04	2.74 (1.4–7.96)	**0.05**
	Moderate	77 (69.4%)	193 (78.5%)	0.96 (0.32–2.81)	0.11	0.67 (0.12–2.46)	0.15
	Strong	5 (4.5%)	12 (4.9%)	1		1	

AOR, adjusted odds ratio; COR, crude odds ratio; CI, Confidence interval; Sig, Significance 0.

## Discussion

This study was the inaugural assessment of problematic test anxiety linked to the newly introduced national exit exam program for prospective graduating respondents in Ethiopia. The study revealed that about two-thirds of respondents (68.9, 95% CI, 63.9–73.7%) experienced significant test anxiety, aligning with findings from studies in Saudi Arabia (65%) (Khoshhal et al., 2017) and Kenya (68.1%) (Ndirangu et al., 2009). However, the finding was higher than the study conducted in USA (55%) (University BMETS, 2013), Malaysia (32.6%) (Rajiah et al., 2014), Canada 38.5% (Gerwing et al., 2015), China (33.7%) (Liu et al., 2021), Iran (43.4%) (Asayesh et al., 2016), Sudan (54%) (Bashir et al., 2019), Gondar (54.7) (Hanfesa et al., 2020) and Addis Ababa 52.30% (Tsegay et al., 2019). The difference might be attributable to variations in sample size, methodology, and participant demographics. This includes the utilization of diverse instruments to assess test anxiety, such as the Hamilton Anxiety Rating Scale employed in Sudan, the Westside Text Anxiety Scale used in Malaysia and Addis Ababa, and the Test Anxiety Questionnaire administered in Gondar. Furthermore, the implementation of a new national exit exam program might contribute to elevated stress levels and unfamiliar expectations among graduating students.

In contrast, the study findings were lower than those reported in studies done in India (83%) (Manisha et al., 2019) and UAE (89.4%) (Tassadaq et al., 2016). The observed difference in results could be attributed to variations in the study populations. This study included prospective graduate students from a range of colleges, whereas the studies conducted in India and the United Arab Emirates solely recruited medical students known to experience high course loads. Medical students confront a substantial volume of intricate medical knowledge during their initial training phases. This knowledge has a direct bearing on patient care decisions, potentially leading to critical consequences. Consequently, the pressure to master this complex information and the apprehension of committing errors that could compromise patient wellbeing can be a significant source of anxiety.

### Social support

This study indicated that respondents with poor social support had 2.74 (AOR = 2.74, 95% CI: 4–7.96) times higher likelihood to develop test anxiety as compared to those with strong social support. This finding was consistent with studies conducted in Turkey (Kavakci et al., 2014), Iran (Asayesh et al., 2016), and India (Hyseni Duraku and Hoxha, 2018). Robust social support networks function as a significant buffer against test-related anxiety in higher education students. This effect is achieved through the cultivation of belonging, validation, and encouragement. Additionally, strong social support equips students with effective coping mechanisms for managing stress and anxiety in a healthy manner.

### Psychological distress

This study highlighted that 99.3% of respondents experienced psychological distress which was higher than studies conducted in Gondar (Hanfesa et al., 2020), Addis Ababa (Tsegay et al., 2019), Saudi Arabia (Alamri and Nazir, 2022), and Canada (Saravanan et al., 2014). The implementation of a new national exit exam may be associated with heightened psychological distress among students. It might be due to the timing of the study, which is near to the actual exam time. This distress could be attributed to a confluence of factors, including: intensified pressure to achieve success, unfamiliarity with the exam format and content, inadequate preparation time, and anxieties regarding future academic or professional prospects. To mitigate these potential stressors, schools can employ a multifaceted support system. This system could encompass readily accessible and comprehensive information about the exam, the allocation of sufficient time for focused preparation, the fostering of a supportive learning environment that prioritizes student wellbeing, and the implementation of strategies for the early identification and intervention for students experiencing psychological distress.

### Coping mechanisms

Furthermore, the current study emphasized that respondents with poor coping mechanisms had 3.67 (AOR = 3.67, 95% CI: 2.45–5.67) times more likely to have test related anxiety and consistent with studies conducted in Iran (Badrian et al., 2022). Ineffective coping strategies, such as procrastination, avoidance behaviors, and negative self-denigration, can exacerbate stress and anxiety levels in students. This, in turn, can hinder their ability to focus and perform optimally on examinations. By identifying and replacing these maladaptive coping mechanisms with more constructive strategies, students can potentially enhance their test-taking performance.

### Family educational level

Additionally, this study noted that respondents with parents lacking formal education had 2.84 (AOR = 2.84; 95% CI: 2.12–4.65) times higher likelihood to progress test anxiety, aligning with studies in Nigeria and Gondar (Hanfesa et al., 2020; Chukwuorji and Nwonyi, 2015). This study posits that parental educational background may influence student test anxiety. Students with parents who lack formal education could experience a confluence of factors contributing to test anxiety, including limited exposure to standardized testing environments during childhood, potentially lower parental expectations regarding academic achievement, and a consequent lack of guidance and support in test preparation strategies. Additionally, these students might face financial insecurity within their families, further exacerbating anxieties related to educational performance. Finally, cultural differences in educational emphasis or testing formats could contribute to a sense of unpreparedness and heighten test anxiety.

### Current grade point average

Lastly, the study revealed that respondents with grade point averages (GPAs) of 2–3 (AOR = 3.25, 95% CI: 2.45–6.78) times to experience test anxiety which is in line with a studies done in Iran (Asayesh et al., 2016), Addis Ababa (Tsegay et al., 2019) and Saudi Arabia (Alamri and Nazir, 2022). Lower grade point averages (GPAs) may be associated with increased test anxiety in students. The relationship between low-grade point average and test anxiety is bidirectional. This phenomenon could be explained by a confluence of factors, including diminished academic self-efficacy stemming from past performance, the ineffectiveness of current study strategies, a history of test failures that create negative associations with testing situations, and the resulting fear of experiencing further academic setbacks. Moreover, test anxiety can also results in poor performance on exams and assignment.

### Limitations of the study

A potential limitation of this study lies in its generalizability and cross-sectional nature (making it difficult to show a cause-and-effect relationship). The research employed a community-based cross-sectional design, collecting data from students at a single university (Dilla University) within a specific timeframe (August 15–September 14, 2023). This restricts the findings to this particular student population during that period. Further research with a larger, more diverse sample across multiple universities throughout the country would be necessary to determine the prevalence and influencing factors of test anxiety for graduating students on a national scale. The use of the Oslo Social Support Scale (OSSS-3) has a relatively low Cronbach’s alpha (0.64), which might affect the reliability of the social support measure.

## Conclusion

This investigation examined the association between a newly implemented national exit exam and test anxiety in graduating students. The results indicated a concerning high prevalence of test anxiety, with roughly 68.9% of participants reporting significant levels. Notably, the study revealed a vulnerability factor: students lacking social support demonstrated a greater propensity for test anxiety. Furthermore, the pressure to succeed and the inherent uncertainties associated with graduation appeared to contribute to a high prevalence of psychological distress (99.3%) among participants. The analysis further identified potential risk factors for test anxiety, including poor coping mechanisms, limited parental education, and lower GPAs. Lower GPAs were likely linked to academic confidence issues and a heightened fear of failure.

Recommendation in light of the findings from this study, a multifaceted approach is recommended to address and reduce test anxiety associated with the new national exit exam among graduating students. Educational institutions should prioritize the development of robust social support networks, particularly for students who lack such support systems, as a buffer against test anxiety. Additionally, the accessibility of counseling services and mental health support programs could directly benefit those respondents with psychological distress identified in the study. It is better to provide cost effective psychological preparedness and coping strategies training by leveraging with existing resource and university community service organizations before the implementation of high stakes exams. Furthermore, efforts to equip students with effective coping mechanisms and educate parents regarding the influence of formal education on test anxiety are warranted. Targeted interventions aimed at boosting academic confidence and mitigating fears of failure among students with lower GPAs could prove beneficial. It is better to conduct further research across multiple institutions to validate the findings and longitudinal studies to explore the temporal relationship between associated factors and test anxiety.

## Data Availability

The original contributions presented in the study are included in the article/supplementary material, further inquiries can be directed to the corresponding author.
